# Implantable cardioverter-defibrillators improve survival after coronary artery bypass grafting in patients with severely impaired left ventricular function

**DOI:** 10.1186/1749-8090-2-6

**Published:** 2007-01-12

**Authors:** Ashraf S Al-Dadah, Rochus K Voeller, Paymon Rahgozar, Jennifer S Lawton, Marc R Moon, Michael K Pasque, Ralph J Damiano, Nader Moazami

**Affiliations:** 1 Department of Surgery, Division of Cardiothoracic Surgery, Washington University School of Medicine in St Louis, St Louis, USA

## Abstract

**Objective:**

Patients with severe left ventricular (LV) dysfunction have a poor long term survival despite complete surgical revascularization. Recent data suggests that the use of Implantable Cardioverter-Defibrillator (ICD) improves survival in patients with severe LV dysfunction. We compared the survival impact of ICD implantation in patients with severe LV dysfunction who underwent CABG.

**Methods:**

Between January 1996 and August 2004, 305 patients with LV ejection fraction (EF) ≤25% had CABG surgery at our institution. Demographics of patients who had received an ICD (ICD+) in the post -operative period was compared to those without ICD (ICD-). Survival was evaluated by the Kaplan-Meier method.

**Results:**

Of the entire group, 35 (11.5%) patients received an ICD with a median of 2 (+/-2) years after CABG. Indication for ICD implantation was clinical evidence of non sustained ventricular tachycardia (NSVT). There were no differences between the 2 groups with respect to age, gender, NYHA classification, number of bypasses, or other co-morbidities. Survival at 1, 3 and 5 years was 88%, 79%, and 67% for the ICD- group compared to 94%, 89% and 83% for the ICD+ group, respectively (figure, p < 0.05).

**Conclusion:**

Implantation of ICD after CABG confers improved short and long term survival benefit to patients with severe LV dysfunction. Prophylactic ICD implantation in the setting of severe LV dysfunction and CABG surgery should be considered.

## Background

Coronary artery bypass grafting in patients with severe left ventricular dysfunction has been an accepted method of treatment for revascularization [1,2]. Nonetheless, sudden cardiac death remains a significant cause of mortality in patients who undergo coronary artery bypass grafting with poor left ventricular function [3]. The use of implantable cardioverter-defibrillator (ICD) devices has been investigated to some extent without conclusive evidence. Insertion of an ICD has proved effective in decreasing the incidence of sudden death in patients with poor left ventricular function in general [4-7]. The CABG Patch trial was a prospective randomized trial evaluating the prophylactic implantation of ICD in patients with ejection fraction (EF) < 36% who underwent CABG. They found no evidence of improved survival among the group of patients with coronary artery disease, depressed left ventricular function and abnormal signal-average electrocardiogram in whom a defibrillator was implanted prophylactically at the time of elective CABG [8]. The CABG-Patch trial had selected patients on the basis of signal-averaged ECG, a marker that may not be as sensitive for predicting sudden cardiac death in patients with severe left ventricular dysfunction. The aim of this study was to investigate the effect of ICD implantation on survival of patients with EF ≤ 25% who underwent CABG, with no previous criterion for selection, except perioperative occurrence of ventricular tachyarrhythmias.

## Methods

Between January 1996 and August 2004, 306 patients were identified in our database that had isolated CABG with a pre-operative EF ≤ 25%. The patients were divided into two groups: patients who received an ICD (ICD+) in the post-operative period and those without ICD (ICD-). All patients were included in this analysis including emergent and urgent operations. Only patients who had concomitant transmyocardial laser revascularization (TMR) were excluded from this analysis. Determination of ejection fraction was based on preoperative left ventriculogram, echocardiography, or a nuclear scan when available.

Perioperative risk factors and demographics were determined from the database and supplemented by a detailed chart review. Of the total of 305 patients, 35 received an ICD (ICD+) and constitute the study group and the remainder served as control. Mortality data was obtained from chart review and supplemented from the United States Social Security Death Index database. This study was approved by the institutional review board of our institution.

### Statistical analysis

Data were analyzed using SYSTAT 11 (SYSTAT software Inc., Point Richmond, CA) Continuous data are reported as mean ± standard deviation (SD), and compared using Student's t test. Categorical variables were analyzed using the χ^2 ^test or Fisher's exact tests as appropriate. Group differences were also considered potentially significant at p < 0.05.

Variables with potential for significant difference between the groups according to the univariate analysis were entered as candidate variables in a multivariate stepwise logistic regression analysis. For each element remaining in the multivariate model, a parameter estimate was calculated from which a p value, odds ratios, and 95% confidence interval for the variable were derived.

Actuarial survival rate curves were calculated according to the Kaplan-Meier method and compared using the generalized Wilcoxon test.

## Results

Indication for ICD implantation was clinical evidence of non sustained ventricular tachycardia (NSVT) in all 35 patients. Mean time for insertion of an ICD device from time of operation was 2 years with a median of 1.2 years and a range of 0 to 6.5 years. There were no differences between the two groups based on age, gender, NYHA functional classification, history of diabetes or other pre-existing co-morbidities (Table 1). Ventricular tachyarrhythmias were slightly more prevalent in the ICD+ group pre-operatively (23% vs. 11%) but this did not reach statistical significance (Table 2). Prevalence of other arrhythmias was similar between the 2 groups with atrial fibrillation dominating in the post-operative period (Table 2).

**Table 1:** Patients demographics and co-morbidities.

		**ICD+ (N = 35)**	**ICD- (N = 270)**
Age		64.60 ± 10.06	66.60 ± 11.29
Gender	Female	9 (25.71)	73 (27%)
	Male	26 (74.29)	197 (73%)
EF		21.43 ± 4.30	21.35 ± 4.44
BMI		27.84 ± 5.05	27.80 ± 5.40
Smoking		27 (77.14%)	187 (69.26%)
Renal failure		5 (14.29%)	45 (16.61%)
Hypertension		25 (71.43%)	182 (67.16%)
Pulmonary hypertension		4 (11.43%)	62 (22.88%)
Angina		24 (68.57%)	210 (77.49%)
Diabetes		25 (71.43%)	133 (49.08%)
Peripheral vascular disease		4 (11.43%)	72 (26.67%)

**Table 2:** Incidence of all types of arrhythmias either pre-operatively or post-operatively.

**Arrhythmia Type**		**ICD+**	**ICD-**
Pre-op	Atrial	3 (8.57%)	42 (15.50%)
	Heart Block	0 (0%)	12 (4.43%)
	Ventricular	8 (22.85%)	30 (11.07%)
	Combined	0 (0%)	14 (5.17%)
Post-op	Atrial	9 (25.71%)	86 (31.73%)
	Heart Block	0 (0%)	7 (2.58%)
	Ventricular	1 (2.86%)	15 (5.54%)
	Combined	0 (0%)	0 (0%)

Overall, approximately three-quarters of the patients in each group had a previous history of myocardial infarction, and slightly over half of these patients had a MI within 3 weeks of CABG surgery (Table 3). In all cases, all appropriate targets were revascularized and more than half of the patients had 3-vessel revascularization. There was no difference between the 2 groups in terms of number and type of grafts used (Table 3).

**Table 3:** Coronary pathology among all patients.

		**ICD+ N = 26**	**ICD- N = 228**
**MI**	**Pre-op**	26 (74.29%)	220 (81.18%)
	**Post-op**	2 (5.71%)	2 (0.74%)
**Duration of MI before CABG**	**6–24 hours**	0	8 (3.51%)
	**1–7 days**	9 (34.62%)	85 (37.28%)
	**8–21 days**	6 (23.08%)	47 (20.61%)
	**> 21 days**	11 (42.31%)	88 (38.60%)
**Left main disease > 50% stenosis**		8 (22.86%)	68 (25.19%)
**Number of bypass grafts**	**Single**	5 (14.29%)	12 (4.44%)
	**Double**	10 (29.57%)	65 (12.96%)
	**Triple**	20 (57.14%)	193 (71.48%)

**Figure 1:**
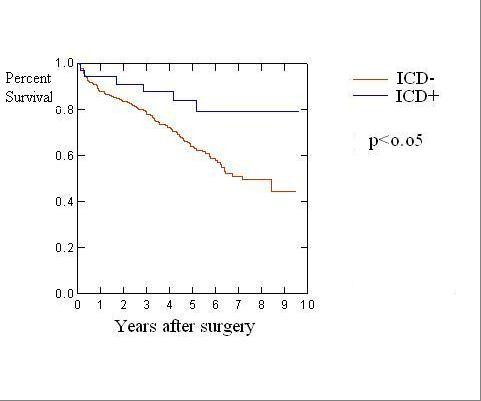
ICD insertion after CABG in patients with severe left ventricular dysfuction improves survival. The Kaplan-Meier Analysis of survival comparing the two group illustrates a significant improvement in survival in patients that received ICD post-operatively vs. patients that did not. Survival percentage is represented on the y-axis and time duration since CABG on the x-axis.

Follow up data for mortality was available for all 305 patients included in the study. The survival benefit for patients with an ICD is illustrated in Figure 1. Survival at 1, 3 and 5 years was 88%, 79%, and 67% for the ICD- group compared to 94%, 89% and 83% for the ICD+ group, respectively (p < 0.05).

**Figure 2:**
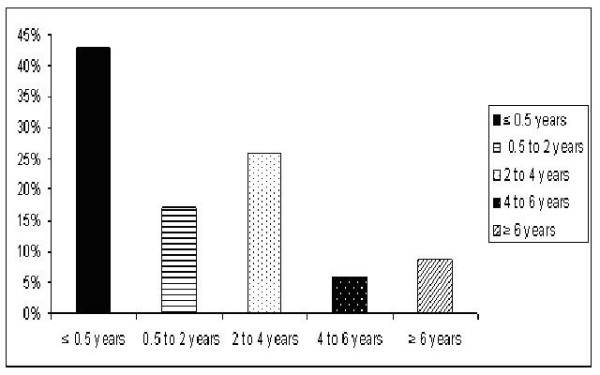
Most patients with ICD's had insertion within the first year after the CABG. This graph illustrates the distribution of patients that received ICD post-operatively with respect to the date of their CABG. The y axis shows the percentage of patients in each group from the total patients that received ICD, the x axis shows the different groups. The groups represented were patients that had an ICD within six months of the CABG, from six months to two years, from two years to four years, from four years to six years and finally over six years from the time of their CABG.

Majority of the patients had their ICD inserted within 6 months of their CABG (Figure 2).

## Discussion

CABG in patients with severe left ventricular dysfunction can be performed with acceptable low operative risk [9-11]. It is associated with good survival and improvement in functional status and ventricular function [10]. Long-term survival nonetheless is hampered with a high incidence of sudden cardiac death [2,3]. Sudden cardiac death (SCD) accounts for 300,000 deaths in the United States, and the incidence of SCD accounts for 0.1–0.2% of all deaths in the general population [12]. When observing a sub-population that has an ejection fraction of < 30%, the percentage rises dramatically and accounts for approximately 20% of all deaths [5-7]. The risk further increases in patients with risk markers for arrhythmias and post-myocardial infarction to approximately 35% [6,13]. The absolute risk of SCD increases with deteriorating left ventricular function and the ratio of sudden to non-sudden deaths is inversely related to the extent of functional impairment [14]. The proportion of sudden death that have an important treatable arrhythmic component is unclear, but trials of implantable defibrillators suggest that this number can be as high as 50% [15,16].

In this retrospective review of patients with severe left ventricular dysfunction, implantation of an ICD in the post-operative period was shown to confer a survival advantage in patients with clinical evidence of non-sustained ventricular tachycardia after coronary bypass surgery. The survival data suggest that the beneficial effects of ICD placement may become evident as early as one year post-operatively. These results mirror what has been already well established in multiple randomized trials.

The investigators of the MADIT I trial studied in a prospective randomized fashion the effect of ICD insertion on patients with previous MI and LVEF < 35% with inducible VT or history of nonsustained VT over medical therapy alone [13]. MADIT II was a prospective randomized study of the effect of ICD insertion on patients with previous MI [5]. They used similar criteria as MADIT I, but without the use of electrophysiologic studies to induce VT. The MUSTT trial was another prospective randomized study evaluating the effect of ICD insertion on patients with LVEF < 40%, coronary artery disease, and history of unsustained ventricular tachycardia with positive inducible ventricular tachycardia [6]. In all 3 studies, ICD insertion resulted in improved survival over conventional medical therapy alone in patients with depressed left ventricular function and coronary artery disease, regardless of the status of revascularization.

In the majority of patients with significant left ventricular dysfunction, it's conceivable that areas of potential ischemic myocardium may remain despite complete surgical revascularization. Many patients have small diseased vessels that are not amenable to bypass surgery. In our patients, over 70% of the patients who had ICD had a history of diabetes. In this difficult patient population, inability to completely revascularize every single area is the rule rather than the exception. In addition, given the high prevalence of previous myocardial infarctions in this patient population, surgical revascularization cannot reverse areas of inhomogeneous scarring. It is our contention that patients with severe left ventricular dysfunction, continue to have the substrate for ventricular tachyarrhythmias despite revascularization. These patients are at risk for SCD from ventricular tachyarrhythmias and early insertion of ICD should be strongly considered. Current rules for reimbursement recommend a waiting period of 3 months after CABG prior to ICD implantation. Whether these recommendations are prudent remains to be seen. Unfortunately, most current thinking is based on the well-designed prospective CABG Patch trial[4]. In this trial no significant improvement of survival was observed with insertion of ICD in patients with low ejection fraction after CABG. The investigators used EF < 36% and abnormal signal-averaged ECG as criteria for patient selection and enrollment. Interestingly, despite no significant reduction in overall mortality between the control group and patients that received ICD, death due to cardiac events was much lower in patients that received ICD post-operatively [17].

Although a retrospective review such as our study has many potential inherent weaknesses and should not be compared to a rigorously performed prospective randomized trial, in our opinion the question remains as to why the CABG Patch trial did not show any significant improvement in survival with ICD. Several plausible explanations may exist.

Ventricular late potentials registered from signal-averaged ECG recordings have been used in risk stratification of patients at risk for sudden cardiac death [18]. Signal-averaged ECG's have been associated with a high negative predictive value and high sensitivity [19,20]. Nonetheless, when observing a sub-population that underwent CABG, signal-averaged ECG did not predict sudden cardiac death [12].

In addition, ventricular late potentials have been recorded in 70–90 percent of patients with spontaneous sustained and inducible ventricular tachycardia after myocardial infarction, in only 0–6 percent of normal volunteers, and in 7 to 15 percent of patients after myocardial infarction that do not have ventricular tachycardia [12]. The presence of these late potentials is a sensitive, but not specific, marker of arrhythmic risk and thus its prognostic use is limited [21]. More recently, Scharf and colleagues found no significance in using signal-averaged ECG in predicting sudden cardiac death in patients that undergo CABG. In their study, the only predictor appeared to be age and low ejection fraction [22].

Finally, the existence of more modern technology in manufacturing of ICD devices may have been a reason for the difference in results between our series and the results of the CABG Patch trial. The fact that the newer generation ICD devices are less implicated in malfunction as opposed to older generations may have yielded improved results in our series. Variability of device manufacturing may also play a role in the altering the results of the CABG Patch trial [23].

Our patient population included all patients with lower ejection fraction than those enrolled in the CABG Patch trial. Indication for ICD insertion was clinical evidence of ventricular tachyarrhythmias. It is possible that many patients who did not receive an ICD either experienced SCD or had episodes of ventricular tachyarrhythmias that were not manifested or recognized clinically. Unfortunately, we were unable to determine the cause of death in the patients reported herein. Nonetheless, with the inherent limitations in this retrospective analysis, the survival benefit of ICD in patients with severely dysfunctional ventricles seems striking.

## Conclusion

In conclusion, the results of this study suggest a reconsideration of early ICD implantation in patients with severely depressed left ventricular function that undergo CABG. The question remains if another prospective trial in this challenging patient population is warranted.

## List of abbreviations

ICD: Implantable cardioverter device

CABG: Coronary artery bypass grafting

MI: Myocardial infarction

EF: Ejection fraction

## Authors' contributions

ASA: First author, conception and design, data collection, analysis and interpretation of data, and drafting the manuscript.

PR: Conception, design and data collection, and drafting the manuscript.

RKV: Conception, design and data collection.

JSL: Conception, design and analysis and interpretation of data.

MRM: Conception, design and analysis and interpretation of data.

MKP: Conception, design and analysis and interpretation of data.

RJD: Conception, design and analysis and interpretation of data.

NM: Principle investigator, conception, design, analysis and interpretation of data, and drafting the manuscript.
